# Epidemiological Characteristics of Acute Coronary Syndrome Patients at Ogulin General Hospital over a Ten-Year Period

**DOI:** 10.3390/jcm15093267

**Published:** 2026-04-24

**Authors:** Marijana Pavlovic, Ivana Sovic, Igor Barkovic, Gordana Starcevic-Klasan, Zeljko Jovanovic, Bojan Miletic

**Affiliations:** 1 Department of Clinical Medical Sciences, Faculty of Health Studies, University of Rijeka, V.C.Emin 5, 51000 Rijeka, Croatiabojan.miletic@uniri.hr (B.M.); 2 Department of Basic Medical Sciences, Faculty of Health Studies, University of Rijeka, V.C.Emin 5, 51000 Rijeka, Croatia; 3 Department of Internal Medicine, Clinical Hospital Center Rijeka, Kresimirova 42, 51000 Rijeka, Croatia; 4 Department of Internal Medicine, General Hospital Ogulin, Bolnicka 38, 47300 Ogulin, Croatia

**Keywords:** acute coronary syndrome, epidemiology, rural area

## Abstract

**Background/Objectives**: Cardiovascular diseases remain one of the major public health challenges in Croatia, with coronary artery disease (CAD) as the most prevalent. The uneven development and concentration of healthcare facilities in urban areas suggest that rural regions lag in providing adequate healthcare. **Methods**: This quantitative, retrospective study included 732 patients diagnosed with acute coronary syndrome (ACS) who were treated between 1 January 2014 and 31 December 2023 at Ogulin General Hospital, Croatia. Data were extracted from the hospital information system, and statistical analyses were performed, with *p* < 0.05 considered significant. **Results**: The analysis showed a decreasing trend in hospitalizations of patients with STEMI (Z = −3.574; *p* < 0.001) and an increase in hospitalizations of patients with NSTEMI (Z = 3.124; *p* = 0.002). No seasonal (χ^2^ = 26.33; *p* = 0.238) or gender differences (χ^2^ = 3.348; *p* = 0.188) were observed. A significant association between age and ACS occurrence was observed (χ^2^ = 57.35; *p* < 0.001). The proportion of patients transferred to another health institution for further treatment was low (39.21%), particularly among patients with STEMI (12.89%). **Conclusions**: The results of this study indicate changes in the dynamics of ACS occurrence during the observed ten-year period. The number of hospitalizations is decreasing significantly, with a very low number of transfers of patients with STEMI; at the same time, there is an increase in hospitalizations of patients with NSTEMI. The study did not show statistically significant seasonal or gender differences in the incidence of ACS, while the incidence of NSTEMI increases with the age of the patients. These results emphasize the need for further improvement in the organization of healthcare for patients with ACS in the rural area served by Ogulin General Hospital.

## 1. Introduction

Cardiovascular diseases (CVDs), including coronary artery disease (CAD), remain the leading public health problem worldwide [1,2,3]. In Croatia, CAD continues to be the leading cause of death [4]. Timely and adequate care for patients with acute coronary syndrome (ACS) is particularly important, as early diagnosis and appropriate treatment significantly influence disease outcomes and reduce mortality [5,6]. Despite advances in diagnostic and therapeutic options, many countries continue to experience disparities in healthcare availability between urban and rural areas, as highlighted by Mesmar et al. and Ortega-Reig et al. [7,8]. Rural areas often have limited access to specialized health services, including invasive cardiological procedures, which can affect the quality of care and lead to higher patient mortality, as found by Faridi et al. [9].

This study was conducted in a central rural area of Croatia served by Ogulin General Hospital, covering a population of approximately 40,000 inhabitants [10]. To date, there are no published data on the epidemiological characteristics of CAD, particularly ACS, in this area, representing a significant gap in understanding local health needs. Therefore, based on multi-year monitoring, the main aim of this study was to determine the frequency of ACS and its individual clinical forms as indications for hospitalization in Ogulin General Hospital, to analyze the frequency in relation to patient sex and age, to examine seasonal variations in disease incidence, and to determine the frequency of the emergency transport of patients to other health institutions for invasive cardiac work-up and treatment. The results of this study should contribute to a better understanding of the epidemiological characteristics of ACS in this rural area and provide a basis for further improvements in healthcare for patients with ACS in this area, thus serving as a model for research and, consequently, better quality care for patients in other rural areas.

## 2. Materials and Methods

### 2.1. Participants and Methods

This study included 732 patients with a diagnosis of ACS treated at Ogulin General Hospital between 1 January 2014 and 31 December 2023. All consecutive patients over 18 years of age admitted to Ogulin General Hospital with a confirmed diagnosis of ACS during the study period were considered for inclusion. No selection was made based on clinical presentation, haemodynamic status, or treatment approach. The patients were divided into three age groups: at or under 40 years, 41 to 65 years, and over 65 years. ACS diagnoses were established according to the ESC guidelines in effect at the time of each hospitalization. Myocardial infarction with ST elevation (STEMI) was defined as chest pain or equivalent ischaemic symptoms with persistent ST-segment elevation of ≥1 mm in at least two contiguous leads or new left bundle branch block confirmed by a subsequent rise in cardiac biomarkers. Myocardial infarction without ST elevation (NSTEMI) was diagnosed in patients with ischaemic symptoms and troponin elevation in the absence of persistent ST-segment elevation. Unstable angina (UA) was defined as ischaemic symptoms occurring at rest or with minimal exertion, accompanied by ECG changes suggestive of ischaemia, without a significant troponin rise [11,12,13]. The primary outcome was the distribution of ACS subtypes (STEMI, NSTEMI, and UA) across age groups and by sex. Secondary outcomes included temporal trends in ACS hospitalizations over the ten-year study period, seasonal variation in monthly presentation, and the frequency of patient transfers according to ACS subtype. Patients under 18 years of age and those with incomplete medical documentation that did not allow reliable identification of the diagnosis, form of the disease, or the essential demographic data required for analysis were excluded from the study. Data on the age, sex, year and month of hospitalization, type of ACS, and the number of patient transfers were collected retrospectively from the hospital information system (BIS), anonymised, and entered into an MS Excel spreadsheet.

### 2.2. Statistical Analysis

The normality of the data distribution was tested using the Kolmogorov–Smirnov test. The incidence of each form of ACS over the years and months, as well as the number of transferred patients with ACS over the years and their diagnoses, was tested using the Chi-square trend test. Deviations and effects of association were presented using standardized residuals and Cramer’s V value. Poisson regression analysis was used to examine the trend in the number of patient hospitalizations over a ten-year period and the annual distribution by month, with 95% confidence intervals. A two-tailed statistical analysis was used to examine trends in hospitalizations and seasonality. The distribution of patients with ACS by age and gender was tested using the Chi-square test. The statistical significance level was set at *p* < 0.05, while standardized residuals are reported at the |Z| > 1.96 level. The statistical programmes MedCalc version 19.0.4 (MedCalc Software bvba, Ostend, Belgium), Statistica version 14.1.0.8 (TIBCO Software Inc., San Ramon, CA, USA), and JASP version 0.96.0.0 (JASP, University of Amsterdam, Amsterdam, The Netherlands) were used for data processing.

### 2.3. Ethical Aspects

During and after the research, the rights and personal data of the respondents were protected in accordance with the Act on the Protection of Patients’ Rights (Official Gazette 169/04, 37/08) and the Act on the Protection of Personal Data (Official Gazette 103/03-106/12). The research was conducted in accordance with the rules of the Declaration of Helsinki (1964–2013) and was approved by the Ethics Committee of the General Hospital Ogulin (No. 01-2/6) on 31 January 2024.

## 3. Results

**Figure 1:**
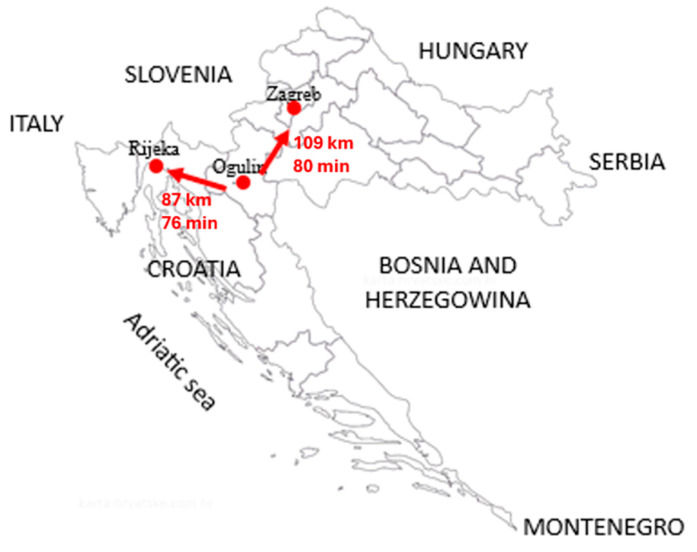
Croatia in Southeast Europe.

This quantitative retrospective study covered the period from 1 January 2014 to 31 December 2023 and included 732 patients treated for a diagnosis of ACS at Ogulin General Hospital, which serves a centrally located area in the Republic of Croatia. Ogulin is equidistant in both time and distance from Rijeka and Zagreb, the nearest centres offering invasive cardiology treatment (Figure 1).

### 3.1. The Trend in Hospitalization of Patients with UA, STEMI, and NSTEMI from 2014 to 2023

**Figure 2:**
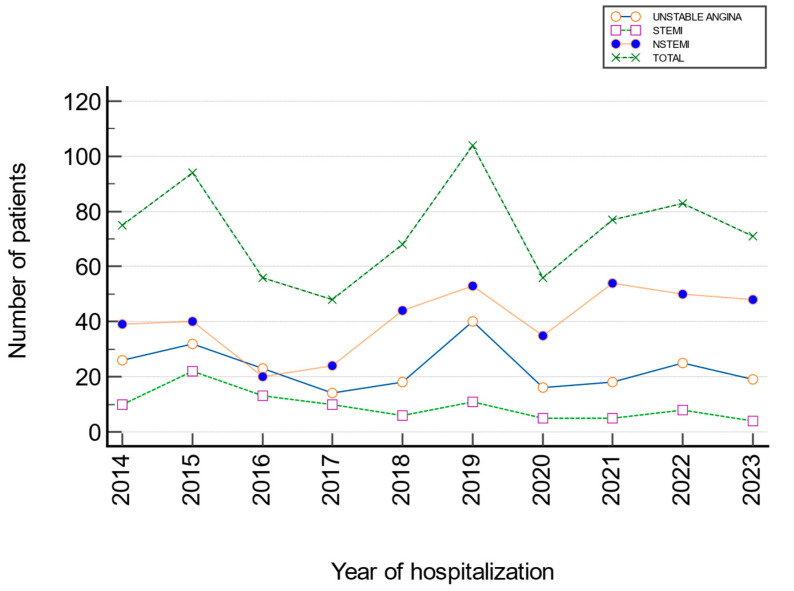
Trend in hospitalization of patients with UA, STEMI, and NSTEMI from 2014 to 2023.

There was a statistically significant difference in the incidence of total ACS across years (χ^2^ = 44.04; Cramer’s V = 0.173; *p* < 0.001). Significant deviations were observed in 2015 and 2016, with a higher proportion of STEMI (Z_2015_ = 3.28; Z_2016_ = 2.41), and in 2021 and 2023, with a higher proportion of NSTEMI (Z_2021_ = 2.71; Z_2023_ = 2.14), while the incidence of UA remained relatively stable during the observed period. Cramer’s V indicates the strength of association (0–0.1 very weak, 0.1–0.3 weak/moderate, 0.3–0.5 moderate/strong, >0.5 strong) (Figure 2, Table 1).

### 3.2. Analysis of the Trend in the Number of Hospitalizations of Patients with UA, STEMI, and NSTEMI During the Period 2014–2023

According to the slope and coefficient values, there was a significant decrease in the number of hospitalizations for STEMI (β_1_ = −0.134; z = −3.574, *p* < 0.001), while there was a significant increase in the number of hospitalizations for NSTEMI (β_1_ = 0.054; z = 3.124, *p* = 0.002) from 2014 to 2023 (Table 2).

### 3.3. Presentation of the Monthly Trend Results for Hospitalization of Patients with UA, STEMI, and NSTEMI

**Figure 3:**
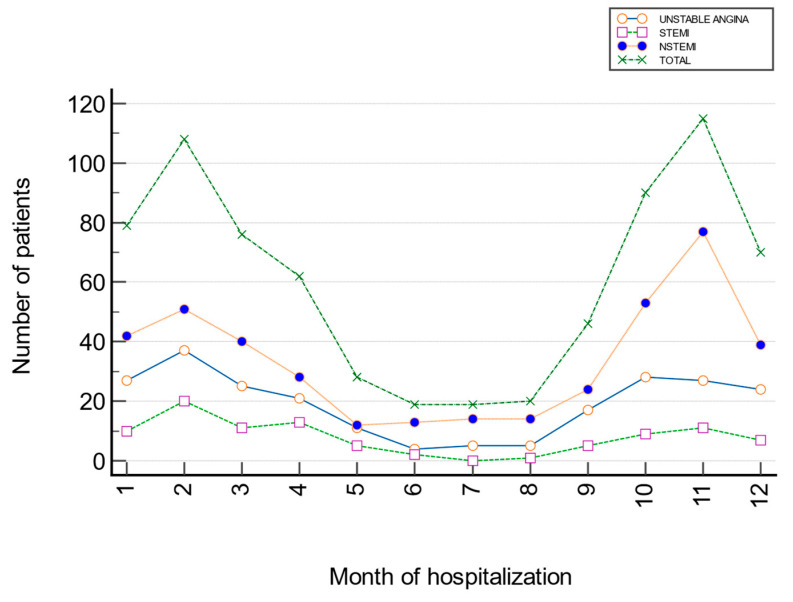
Monthly trend results for the hospitalization of patients with UA, STEMI, and NSTEMI.

There was no statistically significant difference in the incidence of ACS by month (χ^2^ = 26.33; Cramer’s V = 0.134; *p* = 0.238). The standardized residuals showed negative deviations in November for UA (Z_11_ = −2.03), and positive deviations for STEMI in April (Z_4_ = 2.00) and for NSTEMI in November (Z_11_ = 2.67) (Figure 3, Table 3).

### 3.4. Analysis of the Monthly Trend in the Number of Hospitalizations of Patients with UA, STEMI, and NSTEMI Throughout the Year

According to the slope and coefficient values, there was a significant decrease in the number of hospitalizations for STEMI (β_1_ = −0.083; z = −2.697, *p* = 0.007), while a significant increase in the number of hospitalizations for NSTEMI (β_1_ = 0.029; z = 2.001, *p* = 0.045) was observed over the 12 months (Table 4).

### 3.5. Presentation of the Distribution of ACS by Patient Gender

There was no significant difference in the incidence of ACS by gender (χ^2^ = 3.348, df = 2, *p* = 0.188). Cramer’s V = 0.068 indicates a very weak association between gender and the form of ACS. Although the incidence of STEMI and NSTEMI is slightly higher numerically in men, these differences were not statistically significant (Table 5).

### 3.6. Presentation of the Distribution of ACS by Patient Age

There was a significant difference in the age of patients with ACS (χ^2^ = 57.35, df = 4, *p* < 0.001), while Cramer’s V = 0.198 indicated a moderate association between patient age and type of ACS. In the older age group (>65 years), NSTEMI was most prevalent (*n* = 283, 63.31%). In the 41–65 years age group, UA was most common (*n* = 132, 48.00%). In the younger population (≤40 years), the number of NSTEMI cases was higher (*n* = 8, 80.00%) (Table 6).

### 3.7. Analysis of Transferred Patients by Study Year and ACS Type

**Figure 4:**
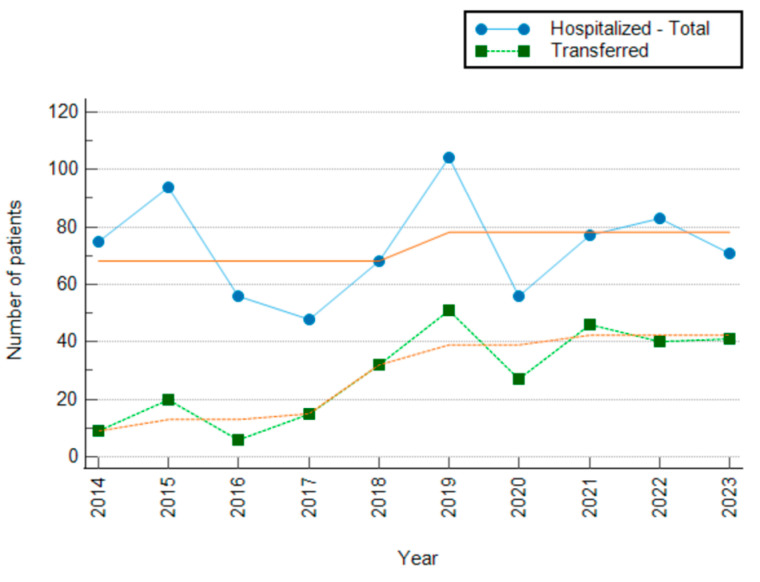
Number of transferred patients by study year.

There was a statistically significant difference in the transfer of patients with ACS across the years in the study period (χ^2^ = 90.87; Cramer’s V = 0.352; *p* < 0.001). Of the total number of patients with ACS (*n* = 732), only 287 were transferred, representing 39.21% of all patients. A significantly reduced percentage of transferred patients was recorded in 2014 (12.00%; Z = −5.09) and 2016 (10.71%; Z = −4.54). During the study period, a positive trend in the number of patient transfers was observed, particularly in 2018 (47.06%; Z = 1.39), and subsequently in 2019 (49.04%; Z = 2.22), 2021 (59.74%; Z = 3.90), and 2023 (57.75%; Z = 3.37) (Figure 4). The value of Cramer’s V indicates a moderate association effect.

There was no statistically significant difference in patient transfer rates by type of ACS (χ^2^ = 3.755; Cramer’s V = 0.072; *p* = 0.153). Of the 39.21% of patients transferred, the lowest transfer rate was observed among STEMI patients (*n* = 37; 12.89%), compared with UA patients (*n* = 102; 35.54%), whereas NSTEMI patients had the highest transfer rate (*n* = 148; 51.57%).

## 4. Discussion

Industrialization and urbanization, along with the resulting population migrations, increase disparities in healthcare between urban and rural areas. As health centres are typically concentrated in urban locations, rural populations often have inadequate access to necessary healthcare, which worsens disease prognosis and outcomes, especially in cases of acute cardiovascular events. Numerous studies have emphasized the disparity in the quality of healthcare between rural and urban populations [14,15,16].

The findings of this study are consistent with the broader international literature documenting these disparities in cardiovascular care. Studies have shown that patients with AMI presenting to rural hospitals have higher mortality rates [9]. Previous research has confirmed that these patients consistently experience longer times to reperfusion, are less likely to receive primary PCI or meet guideline-recommended times to reperfusion, and more frequently receive fibrinolytics than patients living in urban settings [14,17]. The absence of both primary PCI capability and fibrinolytic therapy at Ogulin General Hospital, combined with the low transfer rate observed in this study, places the rural population in this catchment area at a particularly high risk of suboptimal outcomes. Each region has specific sociodemographic, geographical, economic, and cultural characteristics, which should influence the public health policy of that area. The implementation of coordinated regional STEMI networks, based on hub-and-spoke models with clearly defined transfer protocols and reperfusion strategies, has been shown to overcome geographical barriers and ensure timely access to invasive treatment for all patients regardless of location [18].

Our study identified that the incidence of UA did not change over the ten years. At the same time, there was a significant decrease in hospitalizations of patients with STEMI and a significant increase in hospitalizations of patients with NSTEMI. This trend has also been described by other authors, such as Yaser Al Ahmad [19]. In explaining this dynamic, Rogers et al. emphasize that the cause of such trends is the better adherence to clinical guidelines in the treatment of ACS [20]. However, the observed increase in NSTEMI hospitalizations may not solely reflect demographic changes or improved guideline adherence. The progressive introduction of high-sensitivity troponin assays during the study period has substantially lowered the detection threshold for myocardial injury, likely contributing to the reclassification of cases previously categorized as unstable angina to NSTEMI. Data on the exact timing of this diagnostic transition at Ogulin General Hospital were not available; however, this possibility should be considered when interpreting the observed temporal trends in ACS subtypes. In addition, the ageing population leads to more frequent chronic non-communicable diseases, such as CAD.

Regarding the seasonal variability of ACS, our study observed a more frequent occurrence of ACS during the winter months, as also highlighted by Kurihara et al. [21]. The causes of this pattern are still not fully understood. However, it is assumed that one significant factor is cold-induced vasoconstriction, which leads to increased vascular resistance and blood pressure, higher blood viscosity, and a higher tendency for thrombus formation [22,23]. This is further facilitated by the consumption of larger amounts of salt and fat in the diet during colder months [24,25]. Additionally, the more frequent occurrence of various (primarily respiratory) infections, which stimulate the body’s systemic immune response, contributes to the further destabilization of coronary artery plaque and, consequently, a higher incidence of acute coronary events in winter [26]. Our study did not show significant seasonal differences in the incidence of individual forms of ACS, although a slightly higher number of NSTEMI cases was recorded in November and STEMI in April. This distinguishes our findings from those of other studies, such as Baaten et al., which report more frequent plaque rupture (characteristic of STEMI) in winter and plaque erosion and NSTEMI in summer [27]. In our study, when observing the occurrence by month, the number of hospitalizations for STEMI decreased, while the number of hospitalizations for NSTEMI increased.

No statistically significant difference in the incidence of ACS was found between genders, although the incidence of STEMI and NSTEMI in men was higher in absolute terms. This deviates from previous studies, which indicate a higher incidence of CAD in men, especially compared to women of the same age [28,29]. Men are more likely to develop CAD at a younger age due to a higher tendency towards risky behaviour and unhealthy habits and lifestyle compared to women [30]. Hormonal changes during menopause, including a drop in oestrogen, affect vascular function and increase the risk of atherosclerosis and CAD in older women [31].

Considering the age of the patients, NSTEMI predominates in the elderly, while UA is most common in the working population. These findings are consistent with the study by Bhalareo et al., who reported the prevalence of NSTEMI and UA in those over 55 years of age [32]. Several cases of NSTEMI were recorded in the younger population, which aligns with previous findings on the higher prevalence of NSTEMI in young adults compared to STEMI [33].

As invasive cardiac procedures are not performed at Ogulin General Hospital, the analysis also included the transfer of patients with ACS to other health institutions. A statistically significant difference in the transfer of patients with ACS was found during the study period. Of the 732 patients, only 39.21% were transferred to another health institution for further treatment. A particularly low number of transfers was recorded in 2014 (12.00%) and 2016 (10.71%), although an overall positive trend in the number of patient transfers was observed during the study period, especially in 2021 (59.74%) and 2023 (57.75%). Nevertheless, these values remain low compared to current recommendations for the management of ACS, particularly considering that fibrinolytic therapy is not used in prehospital care in the study area [34]. The lowest transfer rate was recorded in patients with STEMI, at 12.89%, and the highest in patients with NSTEMI, at 51.57%.

During the study period, no formal emergency medical services (EMS) protocol existed for the direct transport of STEMI patients to a PCI centre, bypassing Ogulin General Hospital. Hence, all patients with ACS from the catchment area were first admitted to Ogulin General Hospital, where the treating physician decided on transfer. Thus, the reported transfer rates reflect the complete picture of ACS management in this catchment area, with no selection bias introduced by prehospital triage to higher-level facilities. The low transfer rate among STEMI patients (only 12.89%) deserves particular emphasis in the context of current ESC guideline recommendations. As shown in Figure 1, the nearest PCI centres are in Rijeka (87 km, approximately 76 min transport time) and Zagreb (109 km, approximately 80 min transport time). Given that these transport times alone approach the 120 min guideline threshold for first medical contact to reperfusion, and considering the additional time required from symptom onset to hospital arrival and from admission to transfer decision, it is highly probable that most STEMI patients in this catchment area could not have achieved timely primary PCI even if transfer had been initiated promptly. Under these circumstances, the current ESC guidelines recommend fibrinolysis as a bridging reperfusion strategy. However, fibrinolytic therapy was not available at Ogulin General Hospital during the study period, meaning that the overwhelming majority of STEMI patients received no reperfusion therapy. This represents the most critical gap identified in this study and one that likely had direct consequences for patient survival and myocardial recovery; this is strong evidence for the urgent establishment of a regional protocol that either guarantees timely transfer or ensures the availability of fibrinolytic therapy as a bridging measure—a challenge that extends beyond this catchment area and reflects broader inequalities in access to emergency cardiac care across rural regions.

The findings of this study suggest several directions for future research. First, prospective studies incorporating detailed data on in-hospital treatment, including reperfusion strategy, antiplatelet therapy, and time from symptom onset to first medical contact, would allow a more complete assessment of guideline adherence in this setting. Second, the establishment of a regional STEMI registry covering all hospitals within the catchment area, including potential future data on direct EMS transport, would provide a more comprehensive picture of ACS management across the region. Third, outcome data, including in-hospital mortality, MACE, and long-term follow-up after discharge, should be systematically collected to evaluate the clinical consequences of the organisational gaps identified in this study. Finally, the introduction of fibrinolytic therapy at Ogulin General Hospital or the implementation of a formal regional transfer protocol with guaranteed response times should be evaluated prospectively to assess its impact on reperfusion rates and patient outcomes.

### Limitations of the Study

Although this study provides valuable data, it has several limitations. First, its retrospective design and reliance on hospital medical records may have resulted in incomplete or insufficiently detailed data, and the single-centre setting limits the generalizability of the findings to other geographic areas. Although the absence of a direct EMS bypass protocol means the dataset captures all ACS patients admitted from the catchment area, precise timing data—including time from symptom onset to first medical contact and from admission to transfer decision—were not available, precluding a full assessment of guideline adherence regarding reperfusion timelines.

The analysis also did not include several factors that may influence ACS incidence and outcomes, such as detailed comorbidity data, socioeconomic status, and lifestyle habits. Regarding in-hospital management, data on pharmacological treatment—including antiplatelet agents—were not systematically collected. However, fibrinolytic therapy was not performed at Ogulin General Hospital during the study period, meaning that STEMI patients who were not transferred to a PCI centre received no reperfusion therapy. In-hospital outcomes, including mortality, MACE, and bleeding complications, as well as long-term outcomes after discharge, were similarly not assessed.

Additionally, anthropometric data that may be relevant to cardiovascular risk stratification—including chest morphology parameters—were not collected as part of routine clinical documentation and, therefore, could not be included in the analysis [35]. Finally, data on the specific troponin assays used throughout the observation period were not available. The potential introduction of high-sensitivity troponin testing during this period may have influenced the classification of ACS subtypes—particularly the relative proportions of NSTEMI and unstable angina—and represents an additional methodological limitation.

## 5. Conclusions

The results of this study indicate changes in the dynamics of the incidence of ACS during the observed ten-year period, with a significant decrease in the number of hospitalizations of patients with STEMI and an increase in the number of hospitalizations of patients with NSTEMI, while the incidence of UA did not change significantly. The study did not show statistically significant seasonal or gender differences in the incidence of ACS. The incidence of NSTEMI increases significantly with the age of the patients. Importantly, there was a low proportion of patients transferred to institutions that provide invasive cardiac treatment, which indicates organizational challenges in the care of patients with ACS in the rural area that gravitates to the Ogulin General Hospital. The obtained results emphasize the need for further improvement of the organization of health care with the aim of ensuring timely access to modern diagnostic and therapeutic procedures.

## Figures and Tables

**Table 1:** Hospitalization of patients with UA, STEMI, and NSTEMI from 2014 to 2023.

		Year of Hospitalization										
DG		2014	2015	2016	2017	2018	2019	2020	2021	2022	2023	Total
UA	*n*	26	32	23	14	18	40	16	18	25	19	231
	*Z*	0.61	0.56	1.59	−0.37	−0.95	1.64	−0.50	−1.63	−0.30	−0.92	
STEMI	*n*	10	22	13	10	6	11	5	5	8	4	94
	*Z*	0.13	3.28	2.41	1.71	−1.04	−0.75	−0.91	−1.76	−0.93	−1.91	
NSTEMI	*n*	39	40	20	24	44	53	35	54	50	48	407
	*Z*	−0.66	−2.73	−3.12	−0.81	1.59	−1.03	1.08	2.71	0.90	2.14	
Total	*n*	75	94	56	48	68	104	56	77	83	71	732

Legend: DG: diagnosis; UA: unstable Angina pectoris; STEMI: acute myocardial infarction with ST elevation; NSTEMI: acute myocardial infarction without ST elevation; *n*: number of patients; *Z*: standardized residuals.

**Table 2:** Hospitalizations of patients with UA, STEMI, and NSTEMI during the period 2014–2023.

UA	β_1_	S_b_	z	*p*	95% CI
(Intercept) b_0_	60.940	46.328	1.315	0.188	−29.75 to 152.0
Year of hospitalization	−0.029	0.023	−1.248	0.212	−0.074 to 0.016
**STEMI**	**β_1_**	**S_b_**	**z**	*p*	**95% CI**
(Intercept) b_0_	272.918	75.725	3.604	<0.001	126.5 to 424.0
Year of hospitalization	−0.134	0.038	−3.574	<0.001	−0.209 to −0.062
**NSTEMI**	**β_1_**	**S_b_**	**z**	*p*	**95% CI**
(Intercept) b_0_	−105.955	35.101	−3.019	0.003	−175.0 to −37.34
Year of hospitalization	0.054	0.017	3.124	0.002	0.020 to 0.089

Legend: UA: unstable angina pectoris; STEMI: acute myocardial infarction with ST elevation; NSTEMI: acute myocardial infarction without ST elevation β_1_: estimated slope; S_b_: standard error of regression coefficient; z: estimated slope/standard error; b_0_: intercept; CI: confidence interval 95%.

**Table 3:** Month of hospitalization of patients with UA, STEMI, and NSTEMI during the period 2014–2023.

Month of Hospitalization														
DG		1	2	3	4	5	6	7	8	9	10	11	12	Total
UA	*n*	27	37	25	21	11	4	5	5	17	28	27	24	231
	*Z*	0.53	0.65	0.26	0.41	0.90	−1.00	−0.50	−0.64	0.81	−0.10	−2.03	0.52	
STEMI	*n*	10	20	11	13	5	2	0	1	5	9	11	7	94
	*Z*	−0.05	1.91	0.45	2.00	0.81	−0.31	−1.70	−1.06	−0.41	−0.86	−1.14	−0.75	
NSTEMI	*n*	42	51	40	28	12	13	14	14	24	53	77	39	407
	*Z*	−0.46	−1.90	−0.55	−1.73	−1.38	1.14	1.61	1.31	−0.48	0.67	2.67	0.02	
Total	*n*	79	108	76	62	28	19	19	20	46	90	115	70	732

Legend: DG: diagnosis; UA: unstable angina pectoris; STEMI: acute myocardial infarction with ST elevation; NSTEMI: acute myocardial infarction without ST elevation; *n*: number of patients; *Z*: standardized residuals.

**Table 4:** Monthly trend in hospitalizations of patients with UA, STEMI, and NSTEMI.

UA	β_1_	S_b_	z	*p*	95% CI
(Intercept) b_0_	3.118	0.135	23.015	<0.001	2.846 to 3.377
Month of hospitalization	−0.025	0.019	−1.324	0.186	−0.063 to 0.012
**STEMI**	**β_1_**	**S_b_**	**z**	*p*	**95% CI**
(Intercept) b_0_	2.555	0.198	12.884	<0.001	2.152 to 2.930
Month of hospitalization	−0.083	0.031	−2.697	0.007	−0.143 to −0.023
**NSTEMI**	**β_1_**	**S_b_**	**z**	*p*	**95% CI**
(Intercept) b_0_	3.332	0.110	30.201	<0.001	3.111 to 3.544
Month of hospitalization	0.029	0.014	2.001	0.045	0.0006339 to 0.057

Legend: UA: unstable angina pectoris; STEMI: acute myocardial infarction with ST elevation; NSTEMI: acute myocardial infarction without ST elevation; β_1:_ estimated slope; S_b_—standard error of regression coefficient; z: estimated slope/standard error; b_0_: intercept; CI: confidence interval 95%.

**Table 5:** Distribution of ACS by patient gender.

		Gender		
Diagnosis		Male	Female	Total
UA	N	120	111	231
	Col%	28.99%	34.91%	31.56%
STEMI	N	58	36	94
	Col%	14.01%	11.32%	12.84%
NSTEMI	N	236	171	407
	Col%	57.00%	53.77%	55.60%
Total *n*	N	414	318	732
	Col%	100.00%	100.00%	100.00%

Legend: UA: unstable angina pectoris; STEMI: acute myocardial infarction with ST elevation; NSTEMI: acute myocardial infarction without ST elevation; *n*: number of patients; Col%: column percentage.

**Table 6:** Distribution of ACS by patient age.

		Age Group			
Diagnosis		≤40 y	41–65 y	>65 y	Total
UA	*n*	2	132	97	231
	Col%	20.00%	48.00%	21.70%	31.56%
STEMI	*n*	0	27	67	94
	Col%	0.00%	9.82%	14.99%	12.84%
NSTEMI	*n*	8	116	283	407
	Col%	80.00%	42.18%	63.31%	55.60%
Total	*n*	10	275	447	732
	Col%	100.00%	100.00%	100.00%	100.00%

Legend: UA: unstable angina pectoris; STEMI: acute myocardial infarction with ST elevation; NSTEMI: acute myocardial infarction without ST elevation; *n*: number of patients; Col%: column percentage.

## Data Availability

The data are available upon request from the corresponding author.

## Abbreviations

The following abbreviations are used in this manuscript:

ACS	Acute coronary syndrome
CVD	Cardiovascular diseases
CAD	Coronary artery disease
EMS	Emergency medical services
NSTEMI	Acute myocardial infarction without ST elevation
STEMI	Acute myocardial infarction with ST elevation
UA	Unstable angina pectoris
